# Refined Jianpi Huayu Jiedu Decoction Attenuates TAM-Induced Spasmolytic Polypeptide-Expressing Metaplasia (SPEM) by Modulating LCN2-Associated Mitochondrial Dysfunction

**DOI:** 10.3390/ph19050667

**Published:** 2026-04-24

**Authors:** Chongkai Fang, Sijia Zhang, Peiyao He, Yongheng Lai, Rui Luo, Yunshuo Zhang, Sida Liu, Zichun Xie, Huafeng Pan, Wei Liu

**Affiliations:** 1 Science and Technology Innovation Center, Guangzhou University of Chinese Medicine, Guangzhou 510006, China; f.chongkai@gzucm.edu.cn (C.F.); 20231111065@stu.gzucm.edu.cn (S.Z.); fennihpy@foxmail.com (P.H.); zysxzy@outlook.com (Y.Z.); ida_liu723@hotmail.com (S.L.); 13724733164@163.com (Z.X.); 2 The First Affiliated Hospital of Guangzhou University of Chinese Medicine, Guangzhou 510405, China; 13229502030@163.com (Y.L.); luorui@stu.gzucm.edu.cn (R.L.); 3 Guangdong Clinical Research Academy of Chinese Medicine, Guangzhou 510405, China

**Keywords:** SPEM, LCN2, JHJD, mitochondrial dysfunction

## Abstract

**Background:** Spasmolytic polypeptide-expressing metaplasia (SPEM) is an injury-induced gastric epithelial reprogramming state with limited therapeutic options. Although mitochondrial dysfunction has been implicated in epithelial stress responses, its contribution to SPEM development remains incompletely understood. Traditional herbal decoctions have shown potential in alleviating gastric epithelial injury, yet their underlying mechanisms remain largely unclear. **Purpose:** This study aimed to investigate whether refined Jianpi Huayu Jiedu Decoction attenuates tamoxifen (TAM)-induced SPEM, with a focus on LCN2-associated mitochondrial dysfunction. **Methods:** TAM-induced SPEM models were established in mice and gastric epithelial cells. Histological, molecular, and mitochondrial analyses were performed to evaluate SPEM features and epithelial stress responses. UPLC–MS/MS-based chemical profiling, network pharmacology, transcriptomic analysis, and LCN2 knockdown experiments were integrated to explore the underlying regulatory mechanisms. **Results:** Refined Jianpi Huayu Jiedu Decoction significantly alleviated TAM-induced gastric mucosal injury and suppressed the expression of SPEM-associated markers in vivo and in vitro. JHJD treatment improved mitochondrial function, reduced oxidative stress, and normalized mitochondrial dynamics, accompanied by downregulation of LCN2 expression. Chemical profiling identified multiple bioactive components of JHJD, and integrative analyses combining transcriptomics, network pharmacology, and molecular docking suggested that these components are associated with LCN2-related epithelial stress and mitochondrial regulatory networks. Functional validation further demonstrated that LCN2 knockdown partially recapitulated the protective effects of JHJD on mitochondrial homeostasis and epithelial reprogramming. **Conclusions:** These findings indicate that refined Jianpi Huayu Jiedu Decoction attenuates TAM-induced SPEM in association with restoration of mitochondrial homeostasis and suppression of LCN2-related stress signaling, providing mechanistic insight into early gastric epithelial reprogramming.

## 1. Introduction

Gastric cancer remains a major global health challenge, with nearly one million new cases diagnosed each year [1]. While its progression is classically described by the Correa cascade, encompassing chronic atrophic gastritis, intestinal metaplasia, and dysplasia, increasing evidence suggests that gastric epithelial injury and remodeling involve more dynamic and heterogeneous biological processes [2]. Identifying early, reversible epithelial changes indicative of disrupted homeostasis is therefore critical for developing effective intervention strategies [3,4]. Spasmolytic polypeptide-expressing metaplasia (SPEM) is increasingly recognized as a distinct, highly plastic epithelial reprogramming state induced by parietal cell loss and mucosal injury, not a fixed stage within the classical Correa cascade [5]. It is characterized by dedifferentiation of mature epithelial cells and activation of inflammatory and stress-associated signaling pathways, representing an adaptive response to injury rather than irreversible structural alteration, thereby offering a unique window to probe the upstream regulatory mechanisms of early epithelial imbalance [6].

SPEM formation involves aberrant activation of several stress-responsive pathways, including STAT3/LCN2, YAP, and NF-κB signaling, which collectively reshape the epithelial microenvironment and sustain maladaptive repair responses [7,8,9]. Given the energy-intensive nature of epithelial reprogramming, mitochondrial dysfunction and metabolic imbalance may play central roles in initiating and maintaining SPEM. However, the upstream regulators connecting epithelial stress signaling to mitochondrial impairment remain incompletely defined. Lipocalin-2 (LCN2) is a stress-inducible mediator involved in epithelial injury and disease progression via iron handling and redox regulation that disrupts mitochondrial homeostasis and promotes oxidative stress [10]. Elevated LCN2 expression occurs in gastric epithelial remodeling and correlates with inflammation and cellular stress responses [9]. Despite these observations, direct mechanistic evidence elucidating how LCN2 orchestrates mitochondrial dysfunction specifically within the context of SPEM is still lacking, representing a critical gap in understanding early epithelial stress regulation.

Traditional Chinese medicine (TCM) offers multi-target modulation of complex pathological processes such as mitochondrial and redox imbalance, which are central to early gastric remodeling [11,12]. Jianpi Huayu Jiedu Decoction (JHJD), derived from the classical Si Jun Zi Decoction, has demonstrated protective effects in experimental models of gastrointestinal injury and has shown promise in modulating immune responses, inflammatory pathways, and oxidative stress [13]. The formulation was further refined herein to align with early epithelial injury and the reversible nature of SPEM. However, future research may involve updating the name to better reflect the refined formulation. However, whether this refined formulation mitigates SPEM through modulation of LCN2-associated mitochondrial dysfunction remains unclear.

To address this question, we used a TAM-induced SPEM model to investigate mitochondrial dysfunction in early gastric epithelial reprogramming and to determine whether refined JHJD exerts protective effects on the LCN2–mitochondrial stress axis. Integrating in vivo and in vitro experiments with chemical profiling and multi-omics analyses, this work aims to elucidate early epithelial stress regulation and explore potential mitochondrial-targeted intervention strategies.

## 2. Results

### 2.1. JHJD Attenuates TAM-Induced Gastric Injury Without Evident Hepatic or Renal Toxicity

**Figure 1:**
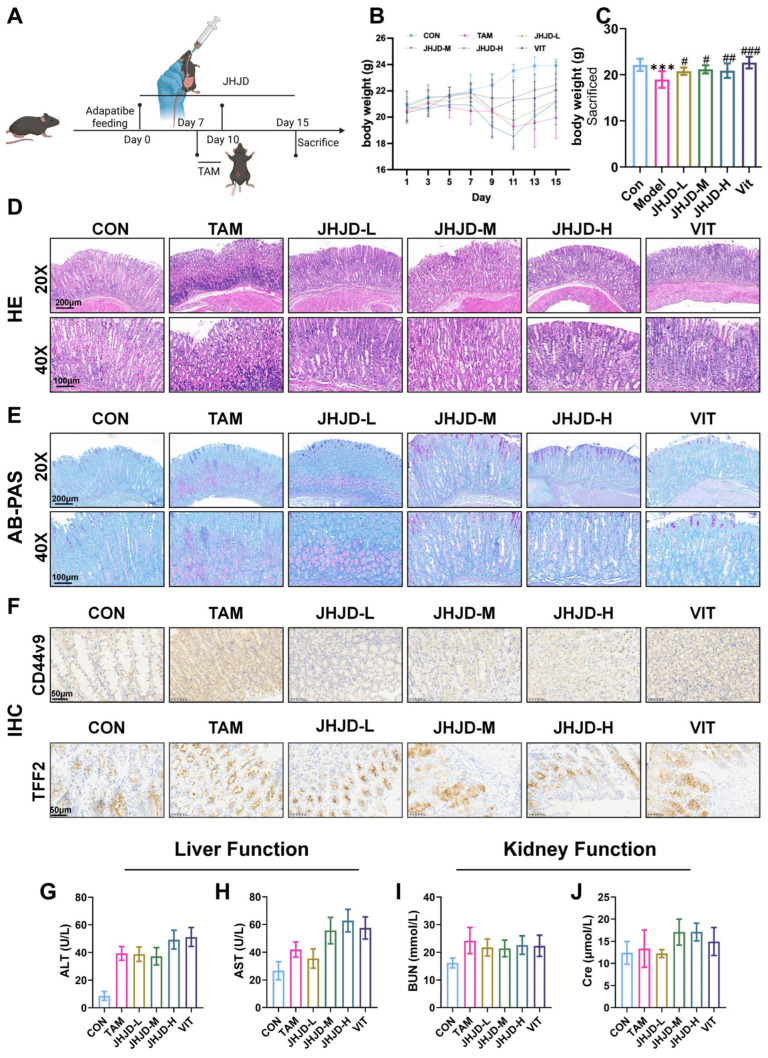
**Establishment and validation of the TAM-induced SPEM model.** (**A**) Schematic overview of the animal experiment design. (**B**,**C**) Changes in body weight of mice. (**D**–**F**) Representative H&E and AB-PAS staining images of gastric tissues. (**E**) Expression of TFF2 and CD44v9 was determined in mice using immunohistochemistry staining. (**F**,**G**) Liver function in each group of mice. (**G**–**J**) Kidney function in each group of mice. All measured biochemical parameters (ALT, AST, BUN, and Cre) remained within the normal physiological range. ^#^ *p* < 0.05, ^##^ *p* < 0.01, ^###^ *p* < 0.001 versus the TAM group; *** *p* < 0.001 versus the CON group.

To evaluate the protective efficacy and systemic safety of JHJD against TAM-induced gastric injury, body weight changes, gastric histopathology, and hepatic and renal function parameters were assessed. TAM administration significantly reduced body weight compared with controls (*p* < 0.05), whereas JHJD or vitacoenzyme (VIT) treatment attenuated this loss and progressively restored body weight throughout the experimental period (Figure 1B). Histological analysis showed that TAM caused severe disruption of the gastric mucosa, including glandular distortion and inflammatory infiltration, as revealed by H&E staining. In addition, AB-PAS staining indicated a reduction in mucin-secreting cells (Figure 1C,D). JHJD treatment, particularly at medium and high doses, effectively preserved mucosal architecture and enhanced mucus production, with effects comparable to those observed in the VIT group. Immunohistochemical analysis further demonstrated that TAM exposure upregulated the SPEM-associated markers CD44v9 and TFF2, whereas JHJD treatment markedly suppressed their abnormal expression (Figure 1E). To assess systemic safety, liver and kidney function parameters were also examined. ALT and AST levels (Figure 1F,G) showed only minor fluctuations among groups, with no significant differences compared with controls (*p* > 0.05). Similarly, BUN and creatinine levels (Figure 1H,I) remained within normal ranges across all groups. These findings indicate that JHJD alleviates TAM-induced gastric injury without causing evident hepatic or renal toxicity.

### 2.2. JHJD Alleviates TAM-Induced SPEM and Mitochondrial Dysfunction in Association with LCN2 Modulation

**Figure 2:**
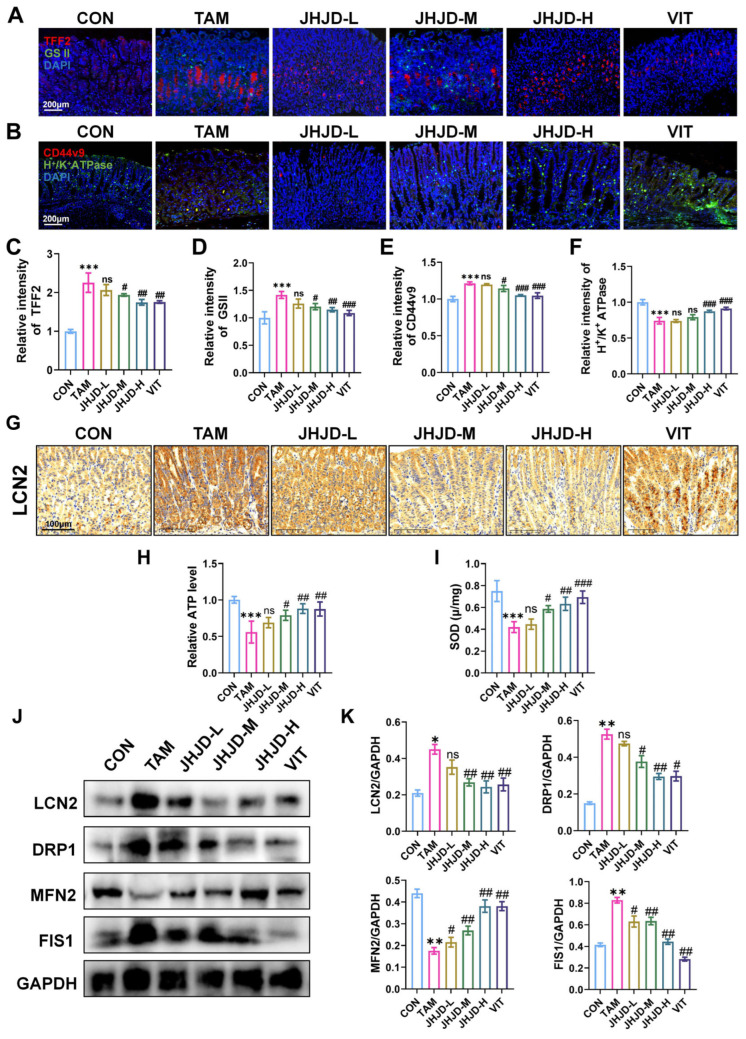
**Histological and molecular characterization of SPEM and the effect of JHJD.** (**A**) Expression of TFF2 and GSII was determined by immunofluorescence with a fluorescence microscope. (**B**) Expression of H^+^/K^+^ ATPase and CD44v9 was determined by immunofluorescence with a fluorescence microscope. (**C**,**D**) Quantitative analysis of mean fluorescence intensity of TFF2 and GSII in each group of cells. (**E**,**F**) Quantitative analysis of mean fluorescence intensity of H^+^/K^+^ ATPase and CD44v9 in each group of cells. (**G**) Expression of LCN2 was determined in mice using immunohistochemistry staining. (**H**) ATP level. (**I**) Serum SOD levels. (**J**,**K**) Western blot and quantification of LCN2, DRP1, MFN2 and FIS1. * *p* < 0.05, ** *p* < 0.01, *** *p* < 0.001 versus the CON group; ^#^ *p* < 0.05, ^##^ *p* < 0.01, ^###^ *p* < 0.001 versus the TAM group.

Immunofluorescence staining revealed that TAM markedly increased the expression of SPEM markers TFF2 and GS II, as well as dedifferentiation-related markers CD44v9 and H^+^/K^+^-ATPase, compared with controls. JHJD treatment significantly (*p* < 0.05) reduced TFF2, GS II, and CD44v9 expression while restoring H^+^/K^+^-ATPase levels, indicating attenuation of SPEM and preservation of gastric epithelial differentiation (Figure 2A–F). Immunohistochemistry showed that TAM induced robust upregulation of LCN2 in gastric tissues, whereas JHJD treatment decreased LCN2 expression (Figure 2G,H). Biochemical assays demonstrated that TAM significantly (*p* < 0.05) reduced ATP content and SOD activity, reflecting mitochondrial dysfunction and oxidative stress. JHJD treatment reversed these changes, with medium and high doses exhibiting the most pronounced protective effects (Figure 2I,J). Western blot analysis further confirmed the regulatory effect of JHJD on LCN2 and mitochondrial dynamics. TAM exposure significantly (*p* < 0.05) elevated LCN2, DRP1, and FIS1 expression while decreasing MFN2, indicating enhanced mitochondrial fission and impaired fusion. JHJD intervention normalized these alterations, restoring the balance of mitochondrial dynamics (Figure 2J,K). Collectively, these findings indicate that JHJD alleviates TAM-induced SPEM and mitochondrial dysfunction through reduced LCN2 expression and restoration of mitochondrial homeostasis.

### 2.3. Chemical Profiling of JHJD by UPLC-MS/MS

**Figure 3:**
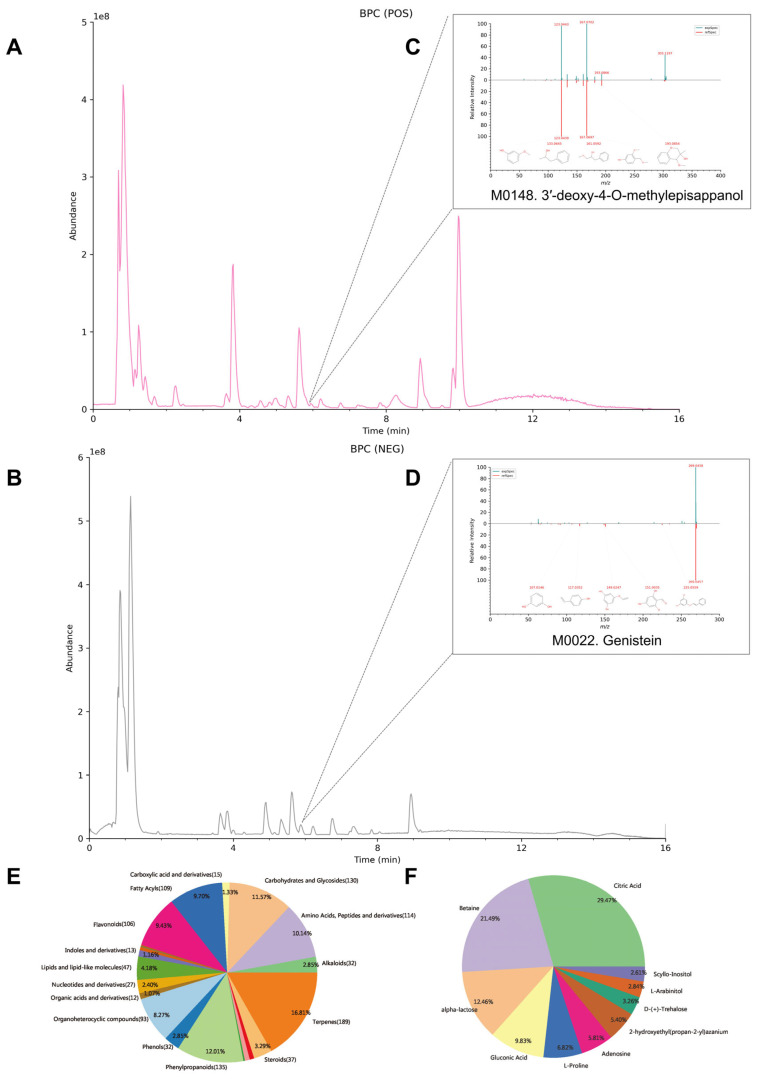
**UPLC-MS/MS profiling and metabolite classification of JHJD.** (**A**) Base peak chromatogram (BPC) in positive ion mode (POS). (**B**) Base peak chromatogram (BPC) in negative ion mode (NEG). (**C**) Representative MS/MS spectrum in positive ion mode. (**D**) Representative MS/MS spectrum in negative ion mode. (**E**) Classification of identified compounds. (**F**) Relative abundance of major metabolites.

To characterize the chemical constituents of JHJD, UPLC-MS/MS analysis was performed under both positive and negative ionization modes. The base peak chromatograms (BPCs) revealed multiple peaks, reflecting the chemical complexity of the formula (Figure 3A,B). Representative constituents identified included 3′-deoxy-4-O-methylepisappanol in positive mode and genistein in negative mode (Figure 3C,D). Component classification analysis showed that the major categories included terpenoids (16.87%), amino acids and peptides (10.14%), carbohydrates and glycosides (11.57%), phenols (12.01%), flavonoids (8.94%), and organic acids (8.27%), among others (Figure 3E). The most abundant individual metabolites were citric acid (29.07%), betaine (21.49%), α-lactose (12.46%), and glutamic acid (9.87%), followed by L-proline and adenosine (Figure 3F). These findings indicate that JHJD contains a broad spectrum of metabolites, with organic acids, amino acids, phenols, and flavonoids being the dominant categories.

### 2.4. Identification of Prototype Components of JHJD in Plasma

**Figure 4:**
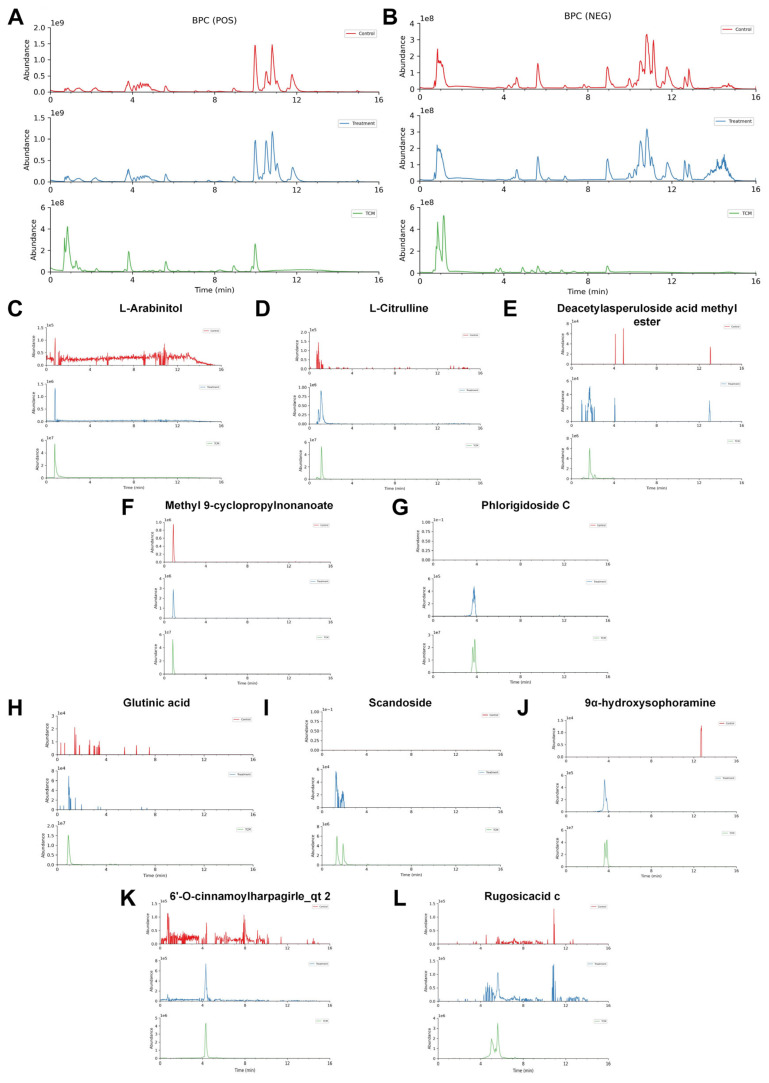
**Comparative UPLC-QTOF-MS/MS analysis of metabolites in control, treatment, and TCM groups.** (**A**) Base peak chromatograms (BPCs) in positive ion mode (POS) for control (red), treatment (blue), and TCM (green) groups, (**B**) BPC in negative ion mode (NEG) for the three groups. (**C**–**L**) Extracted ion chromatograms and MS spectra of representative differential metabolites identified among the groups: (**C**) L-arabinitol; (**D**) L-citrulline; (**E**) deacetylasperuloside acid methyl ester; (**F**) methyl 9-cyclopropylnonanoate; (**G**) phlorigidoside C; (**H**) glutinic acid; (**I**) scandoside; (**J**) 9α-hydroxysophoramine; (**K**) 6′-O-cinnamoylharpagide_qt 2; (**L**) rugosic acid.

To clarify the bioavailable constituents of JHJD, UPLC-MS/MS was used to compare BPCs of blank plasma and plasma collected after oral administration of JHJD. Distinct chromatographic peaks were detected in both positive and negative ion modes, and several prototype compounds were detected in plasma following oral administration of JHJD (Figure 4A,B). Ten representative prototype compounds were identified, including L-arabinitol, L-citrulline, deacetylasperuloside acid methyl ester, methyl 9-cyclopropylnonanoate, phlorigidoside C, glutinic acid, scandoside, 9α-hydroxysophoramine, 6′-O-cinnamoylharpagirle_qt2, and rugosicacid C (Figure 4C–L). These metabolites exhibited clear ion fragmentation patterns, confirming their structural identity. These findings indicate that multiple active constituents of JHJD are absorbed into the circulation, providing a pharmacokinetic basis for its systemic effects.

### 2.5. Network Pharmacology and Molecular Docking Identify LCN2 as a Potential Target of JHJD Against SPEM

**Figure 5:**
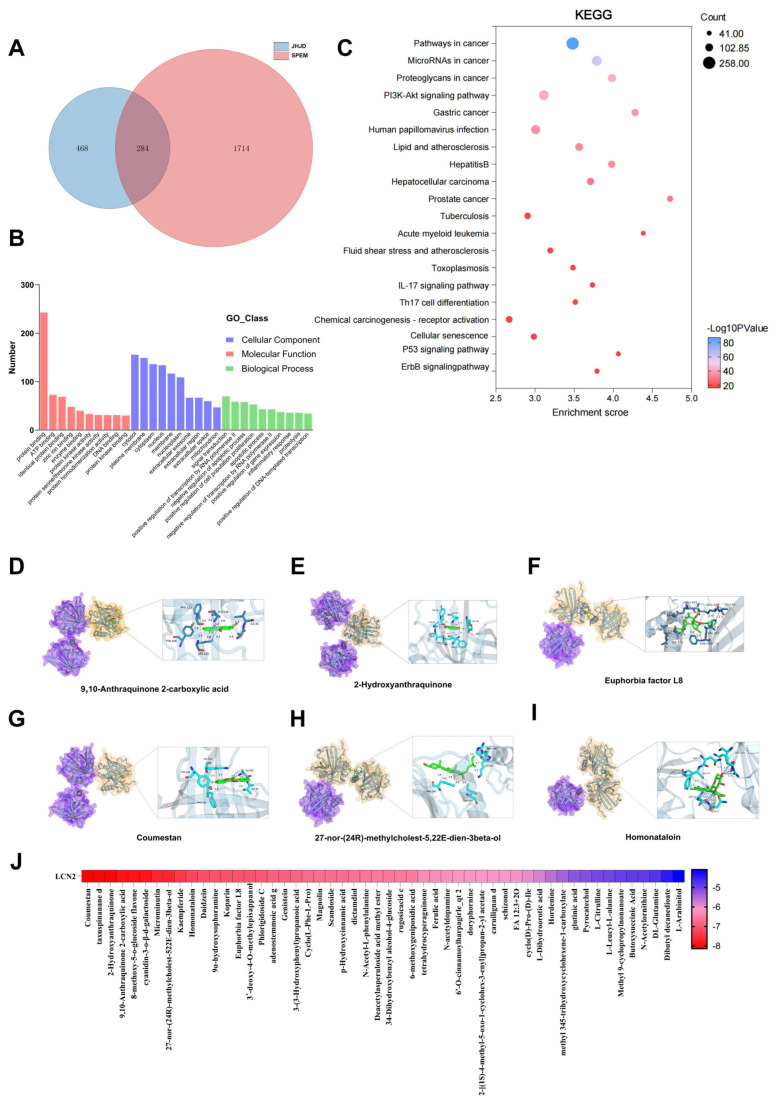
**Network pharmacology and molecular docking analysis of JHJD.** (**A**) Venn diagram showing overlapping and unique differential metabolites between M-JPJD and SPEM groups. (**B**) GO classification of overlapping metabolites: Cellular Component (blue), Molecular Function (red), and Biological Process (green). (**C**) KEGG pathway enrichment analysis. (**D**–**I**) Molecular docking results of representative bioactive compounds with potential target proteins. (**J**) Heatmap of docking scores for top candidate bioactive compounds against LCN2 protein.

Network pharmacology analysis revealed 284 overlapping targets between JHJD-derived blood-entering components and SPEM-related genes (Figure 5A), suggesting that JHJD may exert broad regulatory effects on SPEM. GO enrichment analysis indicated that these targets were mainly associated with cellular stress responses, metabolic regulation, apoptosis, and inflammatory processes (Figure 5B). KEGG pathway enrichment further supported the involvement of signaling pathways related to cellular stress adaptation and epithelial homeostasis (Figure 5C). Although LCN2 was not identified among the predicted targets, it was selected based on its consistent dysregulation in our in vivo and in vitro SPEM models. Network pharmacology analysis was therefore used to assess whether the predicted regulatory landscape of JHJD was compatible with LCN2-associated stress-responsive biological processes, thereby providing system-level support for the subsequent mechanistic investigation. To further evaluate the plausibility of direct interactions between JHJD-derived components and LCN2, molecular docking analysis was performed. Several compounds exhibited favorable binding energies and stable predicted binding modes with LCN2 (Figure 5D–J), suggesting potential molecular interactions that may underlie the regulatory effects of JHJD.

### 2.6. Transcriptomic Analysis Reveals the Regulatory Effects of JHJD on SPEM

**Figure 6:**
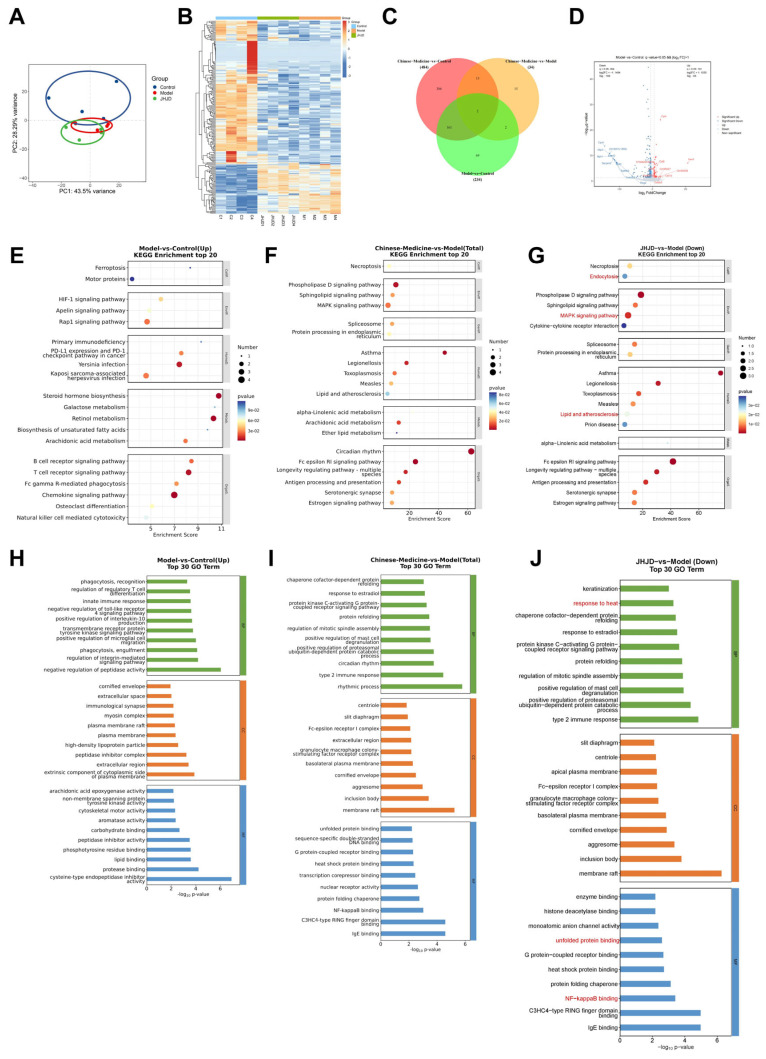
**Transcriptomic profiling of gastric tissues after JHJD treatment.** (**A**) PCA score plot of transcriptomic profiling. (**B**) Heatmap visualization of differentially expressed genes. (**C**) Venn diagram showing overlapping targets of different groups. (**D**) Volcano plot of differential expression analysis for a specific pairwise comparison. (**E**–**G**) Dot plots of the top 10 enriched KEGG pathways for the different groups: (**E**) Model vs. Control (Up), (**F**) Chinese Medicine vs. Model (Total) and (**G**) JHJD vs. Model (Down). (**H**–**J**). Bar plots of the top 30 GO terms in different group comparisons: (**H**) Model vs. Control (Up), (**I**) Chinese Medicine vs. Model (Total), (**J**) JHJD vs. Model (Down).

To further elucidate the global transcriptional alterations associated with TAM-induced SPEM and the regulatory effects of JHJD, transcriptomic profiling was performed on gastric tissues from control, model, and JHJD-treated groups. Principal component analysis (PCA) demonstrated clear separation among the three groups, reflecting pronounced transcriptional reprogramming induced by TAM exposure and partial normalization following JHJD treatment (Figure 6A). Consistently, hierarchical clustering analysis revealed widespread gene expression changes in the model group compared with the controls, many of which were reversed upon JHJD intervention (Figure 6B). The Venn analysis identified overlapping differentially expressed genes (DEGs) among the comparisons, highlighting a subset of transcripts specifically modulated by JHJD (Figure 6C). The volcano plot analysis showed significant (*p* < 0.05) up- and downregulated genes in the model group relative to controls, with a substantial proportion of these alterations restored toward baseline levels after JHJD treatment (Figure 6D). To gain functional insight into these transcriptional changes, KEGG pathway enrichment analysis was conducted. In the model group, DEGs were significantly enriched in pathways related to ferroptosis, HIF-1-related stress signaling, and multiple immune-associated pathways, indicating activation of stress- and injury-associated signaling programs during TAM-induced SPEM. In contrast, JHJD treatment was associated with significant (*p* < 0.05) modulation of pathways involved in necroptosis, lipid and arachidonic acid metabolism, cytokine–cytokine receptor interaction, and other inflammation-associated signaling pathways (Figure 6E–G). Gene Ontology (GO) enrichment analysis further supported these findings. DEGs in the model group were predominantly associated with immune and inflammatory responses, oxidative stress, and apoptosis, whereas JHJD treatment preferentially enriched terms related to protein folding, cellular responses to stress, protein quality control and organelle stress responses, and regulation of immune effector processes (Figure 6H–J). These transcriptomic analyses indicate that JHJD coordinately regulates ferroptosis-, cellular stress response-, protein quality control-, and immune-inflammatory-related gene programs in TAM-induced SPEM, supporting its broad transcriptional regulatory effects on epithelial stress-associated pathway.

### 2.7. JHJD-Containing Serum Suppresses TAM-Induced SPEM Markers In Vitro

**Figure 7:**
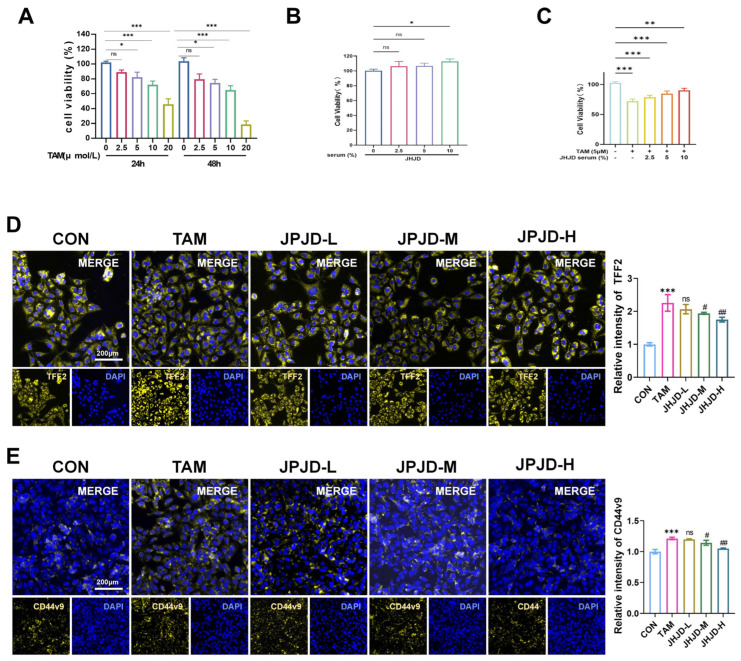
**In vitro SPEM model and effects of JHJD-containing serum.** (**A**) The CCK-8 experiment detected the optimal concentration of TAM. (**B**) The optimal concentration of JHJD-containing medicated serum was determined using the CCK-8 assay. (**C**) Cell viability of GES-1 cells co-treated with TAM and various concentrations of JHJD-containing serum. (**D**) Representative immunofluorescence images and quantification of TFF2 (yellow) and DAPI (blue) staining. (**E**) Representative immunofluorescence images and quantification of CD44v9 (yellow) and DAPI (blue) staining.* *p* < 0.05, ** *p* < 0.01, *** *p* < 0.001 versus the CON group; ^#^ *p* < 0.05, ^##^ *p* < 0.01 versus the TAM group.

The CCK-8 assay was used to determine the optimal concentrations for cell experiments. TAM treatment significantly (*p* < 0.05) reduced cell viability in a dose- and time-dependent manner, with 5 μM selected as the working concentration (Figure 7A). The effects of JHJD-containing serum on cell viability were then evaluated, and 10% serum was chosen as the optimal concentration for subsequent assays (Figure 7B). Co-treatment experiments further showed that JHJD-containing serum alleviated the reduction in cell viability induced by TAM (Figure 7C). Immunofluorescence analysis demonstrated that TAM significantly (*p* < 0.05) increased the expression of the SPEM marker TFF2 and the dedifferentiation marker CD44v9, consistent with the induction of metaplastic changes. Treatment with JHJD-containing serum significantly (*p* < 0.05) reduced the TAM-induced upregulation, with the medium and high concentrations showing the strongest effects (Figure 7D,E). These results indicate that JHJD-containing serum effectively attenuated TAM-induced gastric epithelial metaplastic changes in vitro by suppressing TFF2 and CD44v9 expression.

### 2.8. JHJD-Containing Serum Improves Mitochondrial Function and Regulates LCN2 and Wnt/β-Catenin Signaling In Vitro

**Figure 8:**
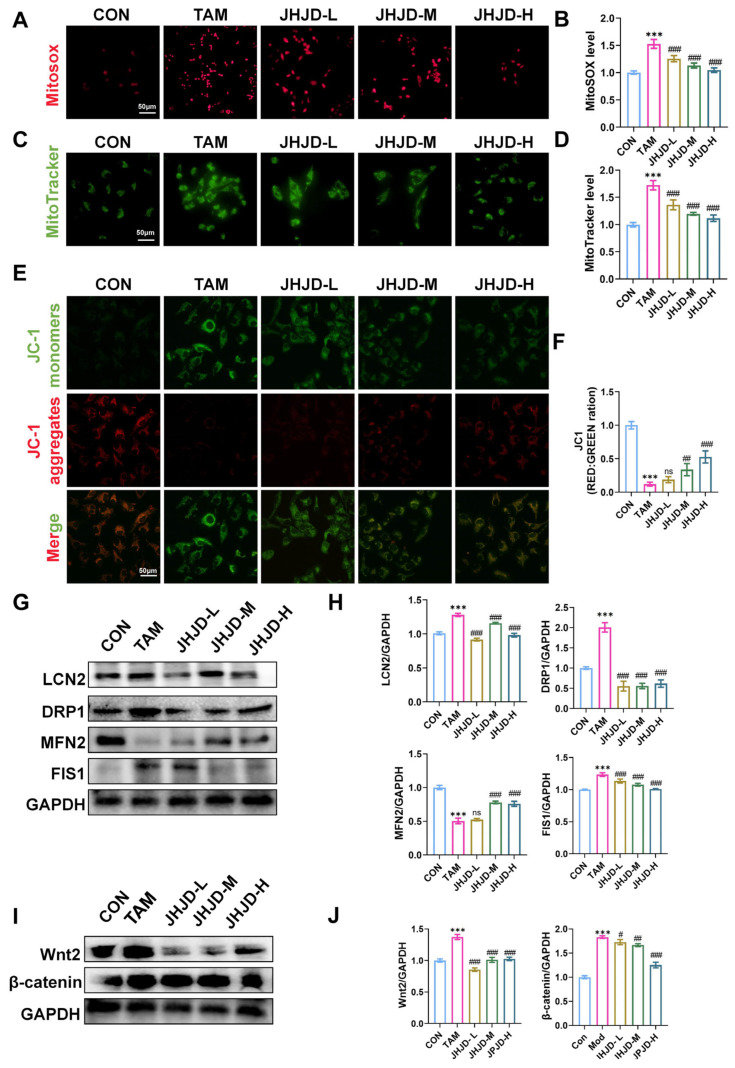
**JHJD alleviates mitochondrial dysfunction and stress signaling in TAM-induced SPEM.** (**A**,**B**) Detection of mitochondrial superoxide changes using MitoSOX Red as a fluorescent probe. (**C**,**D**) Representative immunofluorescence images of MitoTracker Red. (**E**,**F**) Changes in mitochondrial membrane potential detected by the JC-1 probe method. Green: JC-1 monomer; red: JC-1 polymer. (**G**,**H**) Western blot and quantification of LCN2, DRP1, MFN2 and FIS1. (**I**,**J**) Western blot and quantification of Wnt2 and β-catenin. *** *p* < 0.001 versus the CON group; ^#^ *p* < 0.05, ^##^ *p* < 0.01, ^###^ *p* < 0.001 versus the TAM group.

MitoSOX Red staining demonstrated that TAM treatment significantly (*p* < 0.01) increased mitochondrial superoxide levels, indicating enhanced oxidative stress, whereas JHJD-containing serum reduced mitochondrial superoxide accumulation (Figure 8A,B). Similarly, MitoTracker staining showed that TAM markedly impaired mitochondrial integrity, while JHJD-containing serum alleviated this disruption (Figure 8C,D). JC-1 staining further revealed a pronounced loss of mitochondrial membrane potential following TAM exposure, as evidenced by reduced red/green fluorescence ratio. JHJD-containing serum administration significantly (*p* < 0.01) restored mitochondrial membrane potential, with medium and high concentrations showing the strongest protective effect (Figure 8E,F). Western blot analysis confirmed that TAM significantly (*p* < 0.01) induced upregulation of LCN2, DRP1, and FIS1, together with decreased MFN2, reflecting enhanced mitochondrial fission and impaired fusion. JHJD-containing serum treatment normalized these alterations, restoring the balance of mitochondrial dynamics (Figure 8G,H). Given the close association between stress-responsive LCN2 signaling and Wnt/β-catenin-mediated epithelial reprogramming, we next assessed the status of Wnt/β-catenin signaling. TAM exposure significantly (*p* < 0.01) activated Wnt2 and β-catenin expression, whereas JHJD suppressed this aberrant activation (Figure 8I,J). Collectively, these findings indicate that JHJD protects gastric epithelial cells against TAM-induced mitochondrial dysfunction by reducing oxidative stress, restoring mitochondrial dynamics, and modulating LCN2 and Wnt/β-catenin signaling.

### 2.9. Knockdown of LCN2 Alleviates TAM-Induced Mitochondrial Dysfunction and Wnt/β-Catenin Activation

**Figure 9:**
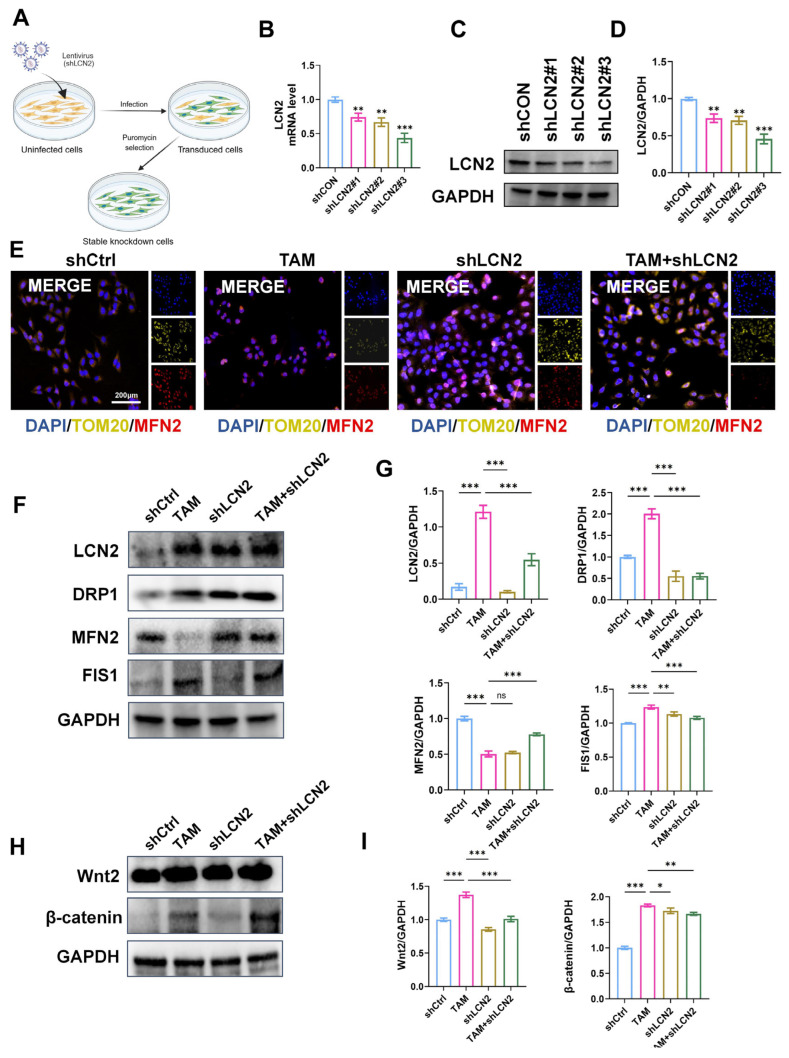
**LCN2 knockdown alleviates mitochondrial dysfunction and stress signaling in TAM-treated gastric epithelial cells.** (**A**) Schematic diagram of the construction of LCN2-knockdown GES-1 cells via lentiviral transfection. (**B**) RT-qPCR analysis of transfection efficiency of different LCN2-targeting lentiviral constructs in GES-1 cells. (**C**,**D**) Western blot analysis of LCN2 protein expression in GES-1 cells transduced with different LCN2-targeting lentiviral constructs. (**E**) Representative immunofluorescence images and quantification of TOM20 (yellow), MFN2 (red) and DAPI (blue) staining. (**F**,**G**) Western blot and quantification of LCN2, DRP1, MFN2 and FIS1. (**H**,**I**) Western blot and quantification of Wnt2 and β-catenin. * *p* < 0.05, ** *p* < 0.01, *** *p* < 0.001 versus the shCtrl group.

To verify the role of LCN2 in SPEM progression, stable LCN2-knockdown GES-1 cells were generated using lentiviral transduction (Figure 9A). RT-qPCR and Western blot confirmed efficient suppression of LCN2 expression by three different shRNA constructs, with shLCN2#2 and shLCN2#3 showing the strongest knockdown efficiency (Figure 9B–D). Immunofluorescence staining revealed that TAM treatment disrupted mitochondrial morphology, characterized by decreased TOM20 and MFN2 signals, whereas LCN2 knockdown markedly preserved mitochondrial integrity. Combined TAM and shLCN2 treatment further confirmed the protective effect (Figure 9E). Western blot analysis showed that TAM significantly (*p* < 0.01) upregulated LCN2, DRP1, and FIS1 expression while downregulating MFN2, indicating mitochondrial fission activation and fusion inhibition. LCN2 knockdown reversed these alterations, restoring mitochondrial dynamics (Figure 9F,G). Furthermore, TAM significantly (*p* < 0.01) activated Wnt2 and β-catenin signaling, whereas silencing LCN2 suppressed this aberrant pathway activation (Figure 9H,I). These findings demonstrate that LCN2 plays a central role in mediating TAM-induced mitochondrial dysfunction and Wnt/β-catenin activation, and that its knockdown confers protective effects against gastric epithelial metaplastic changes.

## 3. Discussion

SPEM represents a highly plastic, stress-responsive state of gastric epithelial reprogramming and is increasingly recognized as a critical and potentially reversible stage preceding intestinal metaplasia and carcinoma [14]. In clinical practice, symptoms frequently precede pathological confirmation, risking the loss of an early, potentially reversible window for intervention. Traditional herbal formulas have been explored as complementary strategies for gastric mucosal injury and metaplastic transformation, but their bioactive basis and mechanistic targets remain poorly characterized [15,16]. In this study, we addressed this gap by integrating chemical profiling, transcriptomic analysis, and functional validation to elucidate the biological pathways underlying SPEM modulation. Our findings demonstrate that JHJD exerts clear protective effects against TAM-induced gastric injury and metaplasia.

Importantly, our data support an LCN2-centered mitochondrial stress axis as a core regulatory framework underlying TAM-induced SPEM. JHJD consistently suppresses LCN2 expression and restores mitochondrial homeostasis, accompanied by attenuation of Wnt/β-catenin activation and reversal of epithelial reprogramming features. This framework positions mitochondrial stress regulation downstream of LCN2 as a primary mechanistic driver of SPEM modulation, rather than a collection of parallel protective effects.

Consistent with this mechanistic framework, anti-SPEM efficacy was substantiated through systematic histological and molecular evaluation. Tamoxifen induced a characteristic pattern of glandular architectural disruption and coordinated lineage reprogramming, characterized by the expansion of TFF2- and GSII-positive metaplastic cells, upregulation of CD44v9, and reduced H^+^/K^+^-ATPase expression. JHJD consistently reversed this hallmark pattern of metaplastic markers in vivo and in epithelial cell models, indicating specific suppression of parietal cell loss-associated epithelial reprogramming rather than nonspecific protection. In parallel, routine liver and kidney function indices remained comparable across groups, suggesting that the observed epithelial benefits were not confounded by systemic toxicity.

Having established a robust anti-SPEM effect, we next explored the potential pharmacodynamic basis underlying this activity. Chemical profiling revealed a diverse array of organic acids, amino acids, phenols, flavonoids, and terpenoids. Several abundant constituents—such as citric acid, betaine, glutamic acid, and adenosine—are closely linked to mitochondrial metabolism, redox regulation, and epithelial recovery [17,18]. While betaine and adenosine are known to regulate stress adaptation and energy balance [19], citric acid and glutamic acid remain largely unexplored in gastric metaplasia. Plasma profiling after intragastric administration in SD rats by UPLC–MS/MS identified circulating prototype and metabolized compounds, supporting systemic exposure and in vivo relevance. Trigonelline, acteoside, and DL-arginine have been reported to mitigate gastrointestinal inflammation and mitochondrial dysfunction, providing mechanistic plausibility for their contribution to JHJD activity [20,21,22]. Collectively, these findings suggest that the multi-component composition of JHJD converges on mitochondrial stress regulation, consistent with the LCN2-centered axis identified in this study.

Mitochondrial dysfunction has emerged as a key driver of epithelial stress responses and lineage plasticity. Gastric epithelial cells are highly sensitive to mitochondrial stress. In our model, tamoxifen consistently disrupted mitochondrial homeostasis. These alterations are consistent with a model in which mitochondrial stress contributes to epithelial reprogramming [23,24]. Treatment with JHJD markedly alleviated these abnormalities, indicating that preservation of mitochondrial homeostasis represents a central mechanism for its anti-SPEM effect. Transcriptomic analyses further revealed enrichment of pathways related to oxidative stress, metabolic regulation, and Wnt/β-catenin signaling, reinforcing a mitochondria-centered interpretation of epithelial reprogramming [25]. Together, these findings reinforce mitochondrial stress regulation as a principal mechanistic axis underlying SPEM modulation in this model.

At the molecular level, LCN2 emerged as a central mediator linking mitochondrial stress to epithelial plasticity during SPEM progression. LCN2 was strongly induced by tamoxifen and markedly reduced by JHJD, paralleling recovery of mitochondrial integrity and epithelial differentiation. In the gastric epithelium, LCN2 is preferentially expressed in proliferative cells, associates with inflammatory signaling, and contributes to invasion and epithelial–mesenchymal transition [26]. In parietal cells, LCN2 promotes ROS accumulation and perturbs iron homeostasis, aggravating mitochondrial dysfunction and accelerating SPEM [9]. Aberrant Wnt/β-catenin signaling has been reported to drive metabolic reprogramming, enhance oxidative stress, and suppress mitophagic clearance, thereby facilitating gastric precancerous progression [27]. In our study, LCN2 upregulation coincided with heightened Wnt/β-catenin activity, suggesting a functionally coordinated stress-responsive axis that favors epithelial dedifferentiation. Although LCN2 occupies a central position within this stress-responsive network, whether it functions as the primary driver or predominantly acts as an amplifier of mitochondrial and Wnt/β-catenin signaling remains to be fully determined. JHJD disrupted this axis by suppressing LCN2, attenuating Wnt/β-catenin activation, and normalizing mitochondrial dynamics, while LCN2 silencing recapitulated the protective effects of JHJD. These findings were confirmed in tamoxifen-treated mice and in human gastric precancerous cell models, supporting the LCN2-Wnt/β-catenin axis as a potentially targetable regulatory node to delay SPEM progression.

Discrepancies in the reported reversibility of SPEM likely reflect differences in experimental models, injury duration, and inflammatory context rather than inconsistencies in the biology itself [28]. The TAM-induced model used here represents an early, injury-driven, and reversible metaplastic state, making it particularly suitable for dissecting stress-dependent reprogramming mechanisms. Our findings should therefore be interpreted within this framework and not directly extrapolated to chronic or intestinalized metaplasia in advanced disease.

This study demonstrates that refined JHJD mitigates TAM-induced SPEM by restoring mitochondrial homeostasis and modulating the LCN2–β-catenin axis. Multi-omics integration identifies LCN2 as a central node linking epithelial stress, mitochondrial dysfunction, and metaplastic remodeling. Several constituents of JHJD, including genistein and coumestrol, showed high predicted binding affinity for LCN2 in silico, suggesting a potential molecular mechanism for molecular interaction. However, these in silico findings are hypothesis-generating in nature and require further biochemical and pharmacokinetic validation. In addition, the present work relies primarily on murine and cellular models, and the relative contributions of individual components require further pharmacokinetic and component–target validation studies. Future investigations should determine whether targeting LCN2-mediated mitochondrial stress can prevent or reverse early gastric metaplasia in clinically relevant settings.

## 4. Materials and Methods

### 4.1. Preparation of Freeze-Dried Powder of JHJD

The herbs used in JHJD were sourced from Guangzhou Zhixin Pharmaceutical Co., Ltd. and authenticated according to the 2020 Edition of the Chinese Pharmacopoeia. The decoction used in this study represents an optimized version of our previously reported decoction, designed for mechanistic investigation of early epithelial injury. The herbs were decocted in water for 45 min, and the resulting extract was concentrated to 1 g/mL, aliquoted, and stored at −80 °C. The gradient elution procedure is detailed in Table 1.

### 4.2. Experimental Animals

Eight-week-old male C57BL/6 mice (20–23 g) were housed under specific pathogen-free (SPF) conditions with free access to food and water under a 12 h light/dark cycle at 20–26 °C and 50–60% humidity. All procedures were approved by the Laboratory Animal Center of Guangzhou University of Chinese Medicine (Approval No. 20240516004; Approval Date: 17 May 2024).

Mice were randomly assigned to a control group (CON, *n* = 10) or a TAM-induced model group (*n* = 50). The SPEM model was established by intraperitoneal injection of tamoxifen (5 mg/20 g; MedChemExpress, Cat# HY-13757A) dissolved in corn oil for three consecutive days. After model establishment, the mice were divided into a model group (MOD) and three treatment groups receiving JHJD by oral gavage at 3.75, 7.5, or 15 g/kg/day for 2 weeks.

At the end of the experiment, mice were fasted for 24 h, anesthetized, and euthanized for collection of blood and gastric tissues.

### 4.3. JHJD Containing Serum Preparation

Twenty male specific-pathogen-free (SPF) Sprague–Dawley (SD) rats (weighing 200 ± 10 g) were housed under SPF conditions with free access to food and water (12 h light/dark cycle, 20–26 °C, 50–60% humidity). All procedures were approved by the Laboratory Animal Center of Guangzhou University of Chinese Medicine (Approval No: 20240718014; Approval Date: 18 July 2024). The rats were randomly assigned to the control group or the JHJD group (5 g/kg/day). The JHJD group was treated daily by oral gavage, whereas the rats in the control group were only administered normal saline for seven consecutive days. On the 7th day, blood was extracted from the abdominal aorta in a sterile environment after administration for 2 h, and separated serum samples were inactivated in a water bath at 56 °C for half an hour and then filtered and stored at −80 °C for the subsequent cell experiment.

### 4.4. Histological Analysis

#### 4.4.1. H&E and AB-PAS Staining

Gastric tissues were fixed in 10% neutral-buffered formalin for 24 h, dehydrated, embedded in paraffin, and sectioned at 4 μm thickness. Hematoxylin and eosin (H&E) staining was performed to evaluate mucosal pathology, and Alcian Blue/Periodic Acid–Schiff (AB-PAS) staining was used to detect mucin-secreting cells. Histological slides were scanned using a Jiangfeng Pathology Whole-Slide Scanner for analysis.

#### 4.4.2. Immunohistochemistry (IHC)

Tissue sections were deparaffinized, rehydrated in graded ethanol, and subjected to antigen retrieval using sodium citrate buffer. Sections were then blocked with 5% normal goat serum containing 0.1% Triton X-100 for 1 h at room temperature, followed by overnight incubation with primary antibodies at 4 °C. The primary antibodies used were LCN2 (1:200), TFF2 (1:200, 13681-1-AP, Proteintech, Wuhan, China), and CD44v9 (1:500, 960-MSM13-P1ABX, ThermoFisher Scientific, Waltham, MA, USA). On the following day, horseradish peroxidase (HRP)-conjugated secondary antibodies were applied, and DAB substrate (P0202, Beyotime, Nantong, China) was used for visualization. Nuclei were counterstained with DAPI. Images were captured using the Jiangfeng Pathology Whole-Slide Scanner.

### 4.5. Immunofluorescence Staining

#### Tissue Sections

Paraffin-embedded gastric tissue sections (4 μm) were deparaffinized in xylene and rehydrated through a graded ethanol series. Antigen retrieval was performed in citrate buffer (pH 6.0) using microwave heating. After cooling to room temperature, sections were washed with PBS and blocked with goat serum (ZSGB-BIO, ZL1-9056) for 60 min to prevent nonspecific binding.

Sections were then incubated overnight at 4 °C with primary antibodies against GSII (1:200, L32451, ThermoFisher Scientific), H^+^/K^+^-ATPase (1:100, sc-374094, Santa Cruz Biotechnology, Dallas, TX, USA), CD44v9 (1:200), and TFF2 (1:200). After washing with PBS, sections were incubated with fluorophore-conjugated secondary antibodies (green, 1:200, E-AB-1055; red, 1:200, E-AB-1075; Elabscience, Wuhan, China) for 1 h at room temperature in the dark. Nuclei were counterstained with DAPI (Beyotime, C1005) for 5 min. Fluorescence images were acquired using a fluorescence microscope (Carl Zeiss, Oberkochen, Germany).

### 4.6. Cultured Cells

GES-1 cells from different treatment groups were seeded onto sterile coverslips in 24-well plates. Cells were fixed with 4% paraformaldehyde for 15 min at room temperature, permeabilized with 0.1% Triton X-100 for 10 min, and blocked with goat serum for 60 min. Cells were incubated overnight at 4 °C with primary antibodies against TOM20 (1:200) and FMN2 (1:200). After PBS washes, cells were incubated with fluorophore-conjugated secondary antibodies (yellow, 1:200, E-AB-1011, Elabscience) for 1 h at room temperature in the dark. Nuclei were counterstained with DAPI. Coverslips were mounted using antifade mounting medium and imaged with a fluorescence microscope (Carl Zeiss, Germany).

### 4.7. Biochemical Assays

Serum levels of superoxide dismutase (SOD), denosine triphosphate (ATP), aspartate aminotransferase (AST), and alanine aminotransferase (ALT) were measured using commercial kits according to the manufacturers’ instructions. The kits used were as follows: SOD (Beyotime, S0101S), ATP assay (A095-1-1, Nanjingjiancheng, Nanjing, China), AST (C010-2-1, Nanjingjiancheng, China) and ALT (C009-2-1, Nanjingjiancheng, China).

### 4.8. Cell Culture and SPEM Model Induction

Human gastric epithelial GES-1 cells were obtained from the Institute of Biochemistry and Cell Biology, Chinese Academy of Sciences (NCACC, CVCL-EQ22). Cells were cultured in Dulbecco’s modified Eagle’s medium (DMEM; Gibco, Carlsbad, CA, USA, Cat# 11875119) supplemented with 10% fetal bovine serum (FBS; Gibco, Cat# 10100147), 100 U/mL penicillin, and 100 μg/mL streptomycin (Gibco, Cat# 10378016), and maintained at 37 °C in a humidified incubator with 5% CO_2_. To induce a spasmolytic polypeptide-expressing metaplasia (SPEM)-like phenotype, GES-1 cells were treated with tamoxifen (TAM) at a final concentration of 10 μM for 24 h.

### 4.9. Cell Viability Assay

GES-1 cells in logarithmic growth phase were seeded into 96-well plates at 5000 cells/well. After attachment, cells were treated with TAM and various concentrations of JHJD for 24 h. Cell viability was measured using Cell Counting Kit-8 (Beyotime, C0037), by adding 10 μL of reagent to each well, incubating for 4 h in the dark, and measuring absorbance at 450 nm. The IC50 was calculated to determine the appropriate concentrations for intervention.

### 4.10. Stable Transfection of LCN2 Knockdown Cells

GES-1 cells were infected with LV-LCN2-RNAi (PSC105905-1, Shanghai GeneChem Co., Ltd., Shanghai, China) lentivirus to establish a stable knockdown cell line. Cells were seeded in 12-well plates (1 × 10^5^ cells/well) and infected at 70–80% confluence according to the manufacturer’s protocol. Stable transfectants were maintained in complete medium under standard culture conditions.

### 4.11. Mitochondrial Membrane Potential Assay (JC-1)

Mitochondrial membrane potential was evaluated using a JC-1 assay kit (C2003S, Beyotime, China). GES-1 cells were incubated with 250 μL of JC-1 working solution for 20 min at 37 °C and then washed with PBS. Red and green fluorescence signals were observed and captured using a fluorescence microscope.

### 4.12. MitoSOX Red Staining for Mitochondrial ROS Detection

Mitochondrial reactive oxygen species (mtROS) levels were assessed using MitoSOX Red (S0061S, Beyotime, China). GES-1 cells were incubated with 5 μM MitoSOX Red in serum-free medium at 37 °C for 10 min in the dark. After washing three times with warm PBS, fluorescence images were immediately acquired using an inverted fluorescence microscope (excitation/emission: 510/580 nm).

### 4.13. MitoTracker Staining for Mitochondrial Visualization

For mitochondrial visualization, the cells were incubated with 100 nM MitoTracker Green FM (C1048, Beyotime, China) in serum-free medium at 37 °C for 30 min in the dark. The cells were then washed three times with warm PBS and imaged using an inverted fluorescence microscope (Leica Microsystems, Wetzlar, Germany).

### 4.14. Western Blotting

Total proteins were extracted from gastric tissues using RIPA buffer containing phosphatase and protease inhibitors. Protein concentrations were measured using a BCA kit (Beyotime, China). Equal amounts of protein were separated by SDS-PAGE and transferred onto PVDF membranes. After blocking with 5% skimmed milk for 1 h, membranes were incubated overnight at 4 °C with primary antibodies, followed by HRP-conjugated secondary antibodies for 1.5 h. Protein bands were visualized using a Bio-Rad gel imaging system and quantified with ImageJ (https://imagej.net). The primary antibodies used were GAPDH (1:2000, 10494-1-AP, Proteintech, China), LCN2 (1:2000, 183853-2-RR, Proteintech, China), DRP1 (1:2000, 12957-1-AP, Proteintech, China), MFN2 (1:2000, 12186-1-AP, Proteintech, China), FIS1 (1:2000, 10956-1-AP, Proteintech, China), WNT2 (1:2000, 27214-1-AP, Proteintech, China), and β-catenin (1:2000, 51067-2-AP, Proteintech, China). The secondary antibody was HRP-conjugated goat anti-rabbit IgG (1:50,000, SA00001-2, Proteintech, China).

### 4.15. RT-qPCR

Total RNA was extracted from gastric tissues using an RNA extraction kit (AG21035, Accurate Biology, Changsha, China) and reverse-transcribed into cDNA using the Evo M-MLV RT Kit (AG11603). Primers were designed based on NCBI GenBank sequences and synthesized by Sangon Biotech (Shanghai, China). Quantitative PCR was performed with the SYBR Green Pro Taq HS Premixed qPCR Kit (AG11759), and relative mRNA expression was calculated using the 2^−ΔΔCt^ method. Primer sequences are listed in Table 2.

### 4.16. Dentification and Quantitative Analysis of Major Components of JHJD by UPLC-MS/MS

To characterize the chemical composition of the herbal extract, lyophilized powder was subjected to UPLC-MS/MS analysis. Chromatographic separation was performed using a C18 column on an ACQUITY UPLC HSS T3 (100 mm × 2.1 mm, 1.8 um), under both positive and negative ion modes. A gradient elution program with solvents A (water + 0.1% formic acid) and B (acetonitrile) was applied. The flow rate was set to 0.35 mL/min, and the injection volume was 0.35 mL/min. The gradient elution procedure is detailed in Supplementary Table S1. MS detection was conducted with a scan range of *m*/*z* 100–1500. Compounds were identified by matching against public databases.

### 4.17. Serum Sample Preparation for UPLC-MS/MS Analysis

Serum samples from rats stored at −80 °C were thawed on ice. An aliquot of 100 μL serum was transferred into a 1.5 mL Eppendorf tube. Then, 400 μL of protein precipitation solvent (methanol–acetonitrile, 2:1, *v*/*v*) containing mixed internal standards (4 μg/mL) was added, followed by vortexing for 1 min. The mixture was subjected to ultrasonic extraction in an ice-water bath for 10 min and incubated at −40 °C for 30 min to enhance protein precipitation. After centrifugation at 12,000 rpm for 10 min at 4 °C, 400 μL of the supernatant was collected and evaporated to dryness under a gentle nitrogen stream. The dried residue was reconstituted in 200 μL of water–methanol–acetonitrile (1:2:1, *v*/*v*/*v*), vortexed for 1 min, and sonicated for 3 min. The solution was then incubated at −40 °C overnight to further remove residual proteins. After centrifugation at 12,000 rpm for 10 min at 4 °C, 120 μL of the supernatant was transferred into an UPLC–MS vial with an insert for subsequent analysis.

### 4.18. Herbal Extract Sample Preparation

To characterize the chemical composition of the herbal mixture, an aliquot of the decoction (~100 μL) was transferred into a 1.5 mL centrifuge tube. Then, 900 μL of water containing mixed internal standards (4 μg/mL) was added and vortexed for 1 min. The mixture was extracted by ultrasonication in an ice-water bath for 60 min and centrifuged at 12,000 rpm for 10 min at 4 °C. The supernatant was diluted twofold with water containing internal standards (4 μg/mL), and 200 μL was transferred into an LC–MS vial for analysis. This step enabled comparative characterization of the original herbal extract and the absorbed serum components.

### 4.19. Serum Metabolomic Profiling

Mouse serum was collected via retro-orbital bleeding. The samples were clotted at room temperature for 30 min, centrifuged at 3000 rpm for 10 min to isolate serum, and stored at −80 °C until analysis. Serum samples from the CON, MOD, and JHJD groups were analyzed by OE Biotech (Shanghai, China) using UPLC-HRMS (Waters/Thermo) and GC-MS (Agilent 7890B–5977B). Differential metabolites were identified based on *p* < 0.05 and |fold change| ≥ 1.5.

### 4.20. Network Pharmacology-Based Analysis

Active compounds of JHJD were screened from the Traditional Chinese Medicine Systems Pharmacology Database (TCMSP) using pharmacokinetic criteria: oral bioavailability (OB ≥ 30%) and drug likeness (DL ≥ 0.18). To improve target prediction reliability, mass spectrometry-identified compounds were integrated with TCMSP targets and experimental datasets. Disease-related genes associated with SPEM were obtained from the NCBI, GEO, and GeneCards databases. Overlapping targets between JHJD and GPL were identified using a Perl script and visualized with Venn diagrams. The compound–target network was constructed using Cytoscape 3.9.2, and protein–protein interaction (PPI) networks were generated using the STRING database (v12.0). GO and KEGG enrichment analyses were conducted to elucidate the biological functions and pathways of potential targets.

### 4.21. Molecular Docking

Molecular docking was performed to verify the interaction between key blood-entering compounds and core target proteins identified from network pharmacology. The 3D structures of target proteins LCN2 (1 x 71.pdb) were obtained from the Protein Data Bank (PDB). Ligand structures were downloaded from PubChem, converted into 3D format using Open Babel GUI, and prepared with AutoDock Tools (1.5.7). Docking simulations were performed using AutoDock Vina (1.2.5), and binding affinities were calculated. The docking poses were visualized using PyMOL (2.6).

### 4.22. Statistical Analysis

All data were expressed as mean ± standard deviation (SD). Statistical analysis was performed using GraphPad Prism (version 9). Comparisons between two groups were conducted using unpaired Student’s *t*-test, and comparisons among multiple groups were performed using one-way ANOVA followed by Tukey’s post hoc test. *p* < 0.05 was considered statistically significant.

## 5. Conclusions

This study demonstrates that JHJD effectively alleviates TAM-induced SPEM by restoring mitochondrial homeostasis and inhibiting LCN2-associated stress signaling. Integrative chemical profiling, transcriptomic analysis, and functional assays identified mitochondrial dysfunction and LCN2 as central regulatory nodes governing early epithelial reprogramming. These findings elucidate a mitochondria-centered mechanism underlying SPEM modulation and highlight LCN2 as a potential therapeutic target for gastric epithelial injury.

## Figures and Tables

**Table 1:** **Detailed information on the herbs used in JHJD**.

Chinese Name	Scientific Name	Part(s) Used	Amount (g)
Huangqi	*Astragalus mongholicus* Bunge	Root	20
Fuling	*Wolfiporia cocos* (F.A. Wolf) Ryvarden & Gilb	Sclerotia	12
Taizishen	*Pseudostellaria heterophylla* (Miq.) Paxt	Roots	20
Baihuasheshecao	*Scleromitrion diffusum* (Willd.) R.J. Wang	Whole herb	15
Hougujun	*Hericium erinaceus* (Bull.) Pers.	Fruit body	10
Ezhu	*Curcuma phaeocaulis* Valeton	Root tuber	9
Baizhu	*Atractylodes macrocephala* Koidz.	Rhizome	10
Sanqi	*Panax notoginseng* (Burkill) F.H. Chen	Root and Rhizome	3

**Table 2:** **Sequence of primers used in RT-qPCR**.

Target Gene	Primer	Sequence (5′–3′)
LCN2	Forward	CTGAATGGGTGGTGAGTGTG
	Reverse	GCTCTCTGGCAACAGGAAAG

## Data Availability

The datasets used and analyzed during the current study are available from the corresponding author on reasonable request.

## Supplementary Materials

The following supporting information can be downloaded at https://www.mdpi.com/article/10.3390/ph19050667/s1: Table S1: The gradient elution program.

## Abbreviations

AB-PAS, Alcian Blue/Periodic Acid–Schiff; DL, drug likeness; GEO, Gene Expression Omnibus; GO, Gene Ontology; H&E, hematoxylin and eosin; IHC, immunohistochemistry; JHJD, Jianpi Huayu Jiedu Decoction; KEGG, Kyoto Encyclopedia of Genes and Genomes; LCN2, Lipocalin-2; mtROS, mitochondrial reactive oxygen species; NCBI, National Center for Biotechnology Information; NF-κB, nuclear factor kappa-light-chain-enhancer of activated B cells; OB, oral bioavailability; PDB, Protein Data Bank; PPI, protein–protein interaction; ROS, reactive oxygen species; RT-qPCR, reverse transcription quantitative polymerase chain reaction; SD, Sprague–Dawley; SPEM, Spasmolytic polypeptide-expressing metaplasia; SPF, specific pathogen-free; TAM, tamoxifen; TCM, Traditional Chinese medicine; TCMSP, Traditional Chinese Medicine Systems Pharmacology Database; UPLC–MS/MS, ultra-performance liquid chromatography–tandem mass spectrometry; UPLC-HRMS, ultra-performance liquid chromatography–high-resolution mass spectrometry.
