# Supplementary material for: Refined Jianpi Huayu Jiedu Decoction Attenuates TAM-Induced Spasmolytic Polypeptide-Expressing Metaplasia (SPEM) by Modulating LCN2-Associated Mitochondrial Dysfunction
Source: Pharmaceuticals (Basel). 2026 Apr 24;19(5):667. doi: 10.3390/ph19050667 (PMC13209456; doi:10.3390/ph19050667)
Supplement: Supplementary file 1 [file pharmaceuticals-19-00667-s001.zip › pharmaceuticals-4235774-supplementary.pdf]

Table S1. The gradient elution program.

Time (min)	A%	B%
0	95	5
2	95	5
4	70	30
8	50	50
10	20	80
14	0	100
15	0	100
15.1	95	5
16	95	5
